# Apparent Recurrence of Hyperparathyroidism following Quadruple Parathyroidectomy

**DOI:** 10.1155/2013/912701

**Published:** 2013-04-23

**Authors:** David S. H. Bell

**Affiliations:** Southside Endocrinology, 3928 Montclair Road, Suite 130, Mountain Brook, AL 35213, USA

## Abstract

Recurrent hyperparathyroidism can occur following quadruple parathyroidectomy and during radical parathyroidectomy and so that future hypoparathyroidism is avoided autotransplantation of parathyroid tissue is often preformed. Herein, I describe a patient who was thought to have recurrent hyperparathyroidism following a quadruple parathyroidectomy based solely on a high intact parathyroid hormone level. The patient neither had clinical symptoms or signs of hyperparathyroidism nor did the laboratory data other than the increased intact PTH level suggest hyperparathyroidism. A more comprehensive history revealed the cause of the elevated intact PTH so that further expensive, invasive, and potentially dangerous investigations were avoided.

## 1. Introduction

Recurrent hyperparathyroidism following extensive parathyroid surgery is not unusual. When all four glands are removed and hyperparathyroidism recurs, the presence of a fifth gland, perhaps situated in the anterior mediastinum, is often suspected [1]. Similarly, with four-gland parathyroidectomy, hypoparathyroidism is expected and to prevent future hypoparathyroidism autotransplantation of parathyroid tissue, usually to the flexor surface of the lower arm, is performed [2]. 

## 2. Clinical Presentation

A 54-year-old white female after being treated with I131 therapy for Graves' disease was discovered to be hyperparathyroid and had had all four hyperplastic parathyroid glands removed. At the time of the quadruple parathyroidectomy, so that future hypoparathyroidism could be avoided, she had an autotransplantation of parathyroid tissue to the flexor surface of left arm. Following surgery, she was well for five years until the sixth year following the radical parathyroidectomy and parathyroid autotransplantation when she developed memory difficulties which progressively worsened, and when she had a dysphagic spell, she was referred to a neurologist. The neurologist found a parathyroid hormone level of 901 pg/mL (upper limit of normal 65 pg/mL) and referred her for assessment of recurrent hyperparathyroidism. 

She had no bone pain, kidney stones, abdominal pain, constipation, dyspepsia, or psychiatric problems. Her serum calcium was 9.8 mg/dL, phosphorous 3.6 mg/dL, chloride 101 mg/dL, and with a chloride/phosphorous ratio 28.2 (normal less than 30). Urinary calcium was 87 mg/24 hours (normal 100–250 mg/24 hours), 25-OH vitamin D was 27.2 ng/mL (normal over 30 ng/mL), and 1-25-OH vitamin D was 69 pg/mL (normal range 18–72 pg/mL). In spite of these normal levels, her intact PTH level was 562 pg/mL. Therefore, other than a high PTH level there were no clinical signs or symptoms or laboratory data to support a diagnosis of recurrent hyperparathyroidism. 

One diagnosis that was considered and could have explained these findings was the development of end-organ resistance to the action of parathyroid hormone which seemed unlikely [3]. A phone call to the patient established that, at both her neurologist's and endocrinologist's laboratories, cubital venesection was performed on the left side which was just above the parathyroid autotransplantation. A repeat assessment of intact PTH drawn from the right cubital vein revealed a PTH level of 28.9 pg/mL (normal range 12–65 pg/mL). The patient was then assured that the high PTH level was due to the site of phlebotomy and that she did not have hyperparathyroidism and that her memory loss and her dysphagic spell were not due to the manifestations of recurrent hyperparathyroidism. 

## 3. Discussion

This patient developed Graves' disease and primary hyperparathyroidism at the same time. Unfortunately, her Graves' disease was treated first with I131 therapy leading to a fibrotic and anatomically incorrect thyroid bed from which to identify and remove hyperfunctioning parathyroid glands. As a result of this and/or the fact that all four glands being enlarged, a quadruple parathyroidectomy with an autotransplantation of parathyroid tissue to the flexor surface of the left forearm was performed [1, 2]. Factitiously elevated levels of parathyroid hormone were found when cubital venesection was performed on the left where the cubital vein was immediately proximal to the site of this parathyroid autotransplant. Other than the elevated parathyroid hormone level, there was no clinical or laboratory evidence to substantiate the diagnosis of recurrent hyperparathyroidism. Phlebotomy performed at a distant site (right cubital vein) revealed that the intact parathyroid level was normal which matched both the clinical finding and the laboratory data. If this simple procedure had not been performed, further expensive and invasive investigations, including surgical neck and/or mediastinal exploration, might have been performed.

In patients with autotransplantation of parathyroid tissue to the upper limb, phlebotomies to assess parathyroid hormone levels should be performed in the contralateral cubital vein so that the erroneous diagnosis of recurrent hyperparathyroidism with its potential for expensive and invasive investigations can be avoided.
